# A dynamic nomogram predicting nosocomial infections in patients after colon cancer surgery

**DOI:** 10.3389/fonc.2025.1528036

**Published:** 2025-02-28

**Authors:** Xue Yao, Shuhui Wang, Anning Lu, Yun Xu, Na Li

**Affiliations:** ^1^ Department of Joint Surgery, Weifang People’s Hospital, Weifang, Shandong, China; ^2^ Department of Infection Prevention and Control, Qilu Hospital of Shandong University, Jinan, Shandong, China; ^3^ School of Nursing, Shandong Second Medical University, Weifang, Shandong, China

**Keywords:** colon cancer, nosocomial infections, prediction, nomogram, model

## Abstract

**Objective:**

Nosocomial infections are one of the severe postoperative complications that compromise perioperative safety in patients with colon cancer. However, there are limited studies on constructing visual risk prediction screening tools for nosocomial infections in these patients. The objective of this study is to construct a nomogram for predicting the risk of nosocomial infections among patients after colon cancer surgery.

**Methods:**

Total 1146 patients after colon cancer surgery were selected and divided into a training set and a validation set. After identifying the most significant predictors through LASSO regression and logistic regression, the model was presented as static and dynamic nomogram. AUC was used to evaluate the discrimination of model. Calibration was evaluated by means of calibration curves. Decision and impact curves were applied to evaluate the clinical validity.

**Results:**

110 patients (9.60%) suffered nosocomial infections following colon cancer surgery. Peak temperature on the second postoperative day, Braden score on the first postoperative day, duration of retention of abdominal drains, ASA class, surgical type and postoperative complications were correlated with nosocomial infections. The nomogram composed of these predictors demonstrated good discrimination, calibration and clinical benefit in both the training and validation sets.

**Conclusion:**

Risk predictors are important breakthroughs for healthcare workers in nosocomial infections prevention and control initiatives. The dynamic nomogram built in this study may be helpful for healthcare personnel to identify the risk of nosocomial infections among patients after colon cancer surgery.

## 1 Introduction

Colon cancer is one of the common malignant tumors of the gastrointestinal tract worldwide. The proportion of colon cancer has been rising over the past few years. The Global Burden of Cancer 2020 reports that there will be more than 1.14 million new cases of colon cancer and more than 570,000 attributable deaths in 2020 (1). At present, many poor lifestyles and dietary structures further contribute to the younger age of onset of colon cancer (2).

The high morbidity and mortality rates of colon cancer have made it one of the diseases of great concern in the field of healthcare. At this stage, the main form of treatment is to remove the tumor by surgery. How to effectively guarantee the perioperative safety of patients has become the focus of healthcare professionals. In particular, prevention of nosocomial infections (NIs) is one of the most vital elements to ensure perioperative safety. Despite the existence of many interventions aimed at reducing the risk of NIs, NIs pose a number of adverse prognostic consequences for patients. Without effective control of NIs, patients may be at risk of continued deterioration and progression to infectious shock, which can ultimately lead to multiple organ dysfunction syndrome. Therefore, it is extremely important to prevent postoperative NIs among patients after colon cancer. NIs cause a significant disease and economic burden on global healthcare systems (3, 4). A study by MacLaurin A showed that the average annual number of NIs in Canada is about 220,000 (5). The situation of NIs in the United States is also not optimistic, as the annual average number of inpatient deaths due to NIs has long exceeded 100,000 (6). At the hospital level, the occurrence of NIs can lead to medical disputes and negatively affect hospitals’ operational capacity (7). At the patient level, NIs increase the severity of the patient’s condition, prolong length of stay, affect the outcome of in-hospital treatment and quality of life (8), and cause anxiety and distress to the patient.

Nomogram is a predictive model constructed on the basis of Logistic regression analysis, which visualizes graphs showing the extent to which each predictor contributes to the effect of the dependent variable (9). The presentations of nomogram includes both static and dynamic forms. Effective risk prediction models are essential aids to assist healthcare professionals in targeting those at high risk for NIs. However, there are few studies that constructed nomogram for predicting the risk of NIs in patients after colon cancer surgery. Given the severity of NIs, the aim of this study was to explore the potential predictors associated with NIs in patients after colon cancer surgery and to construct a dynamic nomogram.

## 2 Materials and method

### 2.1 Design and participants

**Figure 1:**
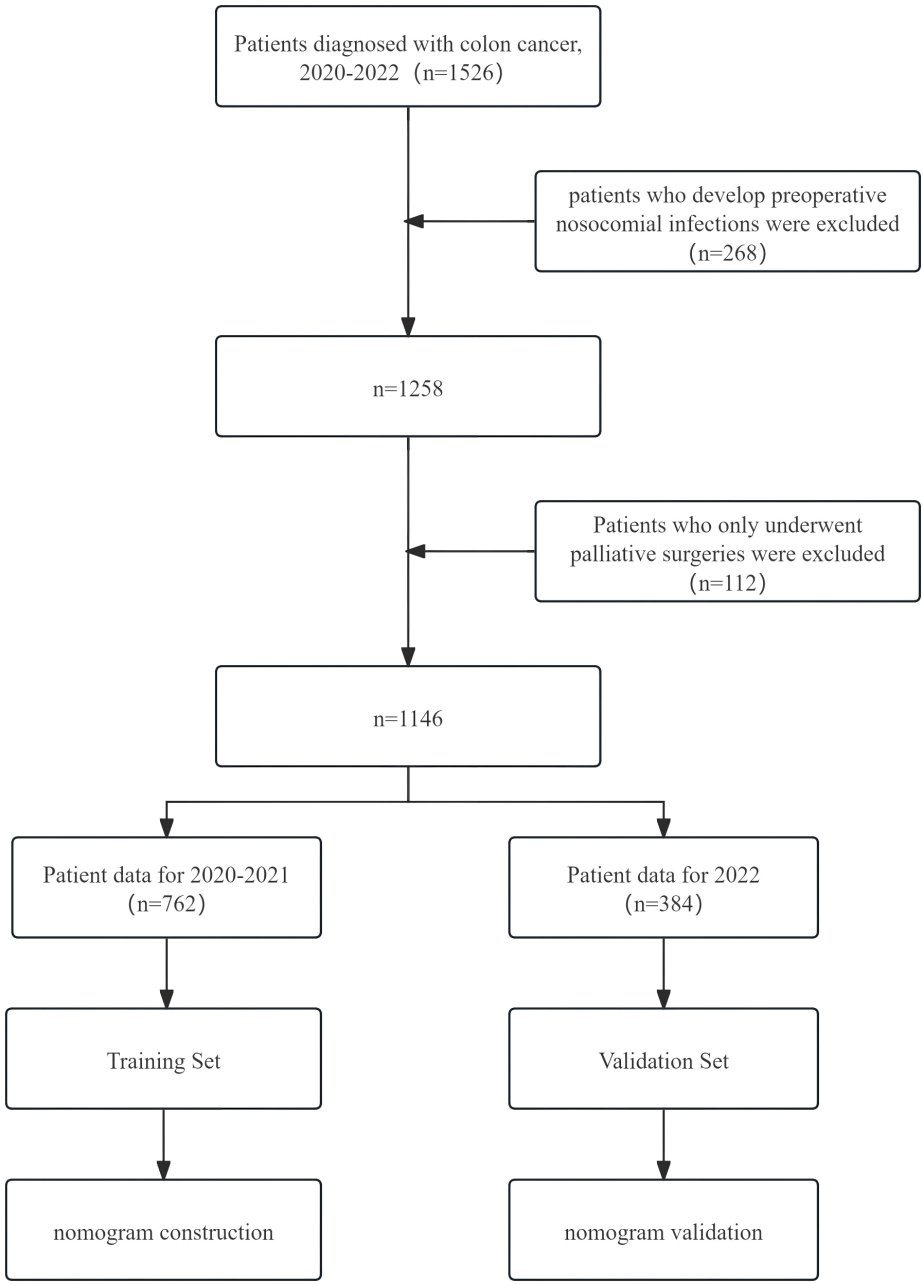
Flow chart of the patient screening and nomogram construction.

In this study, colon cancer patients who underwent surgical treatment between 1 January 2020 and 31 December 2022 in a tertiary comprehensive teaching hospital affiliated to Shandong University were included as study subjects. Patients were included if they were (1) diagnosed with colon cancer, (2) underwent surgery, and (3) were older than 18 years. Patients who only underwent palliative surgeries such as exploratory laparotomy or colostomy, as well as patients who develop preoperative NIs, were excluded. This study was approved by the ethics committee on scientific research of Shandong University and conducted following the Declaration of Helsinki. Due to the retrospective nature of this study and anonymous data collection, informed consent is not required. The patient screening process is shown in **Figure 1**.

Patient data from 2020 to 2021 was used as a training set to construct the model, and patient data from 2022 was used as a validation set to validate the model. In order to achieve a predictive model that accurately estimates the overall risk of occurrence of the outcome event, we used the sample size calculation metric mentioned by Riley et al (10). The formula is as follows:


$$N={\left(1.96/\delta \right)}^{2}\times \varphi \left(1-\varphi \right)$$

The “φ” represents the probability of occurrence of the ending event. The NIs rate of postoperative colon cancer patients in the pre-survey was about 10%, and based on clinical experience, we set the permissible error “δ” to be 0.03, which was calculated to obtain a sample size of 384 cases for the training set.

### 2.2 Diagnostic criteria and surveillance of NIs

Definition of NIS cases according to the diagnostic criteria for NIs issued by the Centers for Disease Control and Prevention (11). Gastrointestinal clinicians made the NIs diagnosis by combining the patient’s clinical symptoms and ancillary test results. To ensure the rigor of the study, all included NIs cases were audited by professional NIs managers.

### 2.3 Data collection

Hospital infection surveillance system and hospital information system were used to complete the collection of clinical data from patients by two independent researchers with professional training. Preoperative and postoperative laboratory values were measured within 24 hours of admission and within 24 hours after surgery, respectively. The postoperative complications that occurred in the patients collected in this study included intestinal obstruction, chyle leak, anastomotic leakage, incision dehiscence, deep venous thrombosis, pericardial effusion, hypokalemia, gastric emptying disability, heart failure, respiratory failure, and fistula of intestine. Since NIs being positive outcome events in this study, they were not included in the postoperative complications mentioned above. All predictor variables were collected prior to the outcome event.

### 2.4 Statistical analysis

We analyzed all data using SPSS version 26.0 and R 4.1.0 software. Categorical variables were statistically described using frequencies, rates or percentages (%). Inter group comparisons were analyzed with Pearson’s chi-square test or Fisher’s exact probability test. Continuous variables were described as mean ± standard deviation (SD) if normally distribution, otherwise as median (interquartile range). Inter group comparisons were analyzed with the Student’s t-test or Mann-Whitney U-test. *P* < 0.05(two-sided) were considered significant.

The least absolute shrinkage and selection operator (LASSO) regression algorithm is an estimation method that enables the streamlining of indicator sets (12). LASSO is based on the principle of adding a penalty term to the least squares basis to compress the estimated parameters, making them zero when they are reduced to less than a threshold, and ultimately producing a set of independent variables with some correlation with the ending variables (12–15). LASSO is suitable for dealing with data that may be subject to collinearity and studies with a low number of outcome events (16). In LASSO regression, the adjustment of model complexity can be achieved by adjusting the value of “λ” (17). In this study, 10-fold cross-validation was used to determine the optimal “λ” value. Due to the large number of variables collected in this study, in order to reduce the impact of collinearity between them, we used LASSO to conduct preliminary screening of the variables. Afterwards, we used Logistic regression for final screening of independent risk predictors of NIs in patients after colon cancer, and finally developed a logic-model based NIs Nomogram. The above method of screening variables is referred to as lasso-logistic regression (18). *P* < 0.05(two-sided) were considered significant.

We used the independent risk predictors to construct a nomogram. Discrimination of the model can be represented by receiver operating characteristic (ROC) curve (19). Discrimination increases gradually as the AUC value approaches 1. We used the Hosmer-Lemeshow test and the calibration curve to evaluate calibration (19). The *P* > 0.05 of the Hosmer-Lemeshow test indicates a good calibration ability of the model. In addition, the clinical benefit of the nomogram in this study was assessed by decision curve analysis (DCA) and clinical impact curve (CIC). DCA can be used to evaluate the potential population impact of applying nomogram in clinical practice. The vertical axis shows standardized net benefit, while the horizontal axis represents the risk threshold. CIC is generated based on DCA. It can display the estimated number of people predicted as high-risk at each risk threshold and intuitively show the proportion of true positive patients (20).

## 3 Result

### 3.1 Characteristics of patients

Ultimately, this study comprised 1146 colon cancer patients who underwent surgery, with an average age of 59.79 ± 12.31 years. The number of patients in the training set was 762, with an average age of 60.11 ± 12.51 years. The number of patients in the validation set was 384, with an average age of 59.16 ± 11.89 years. In this study, a total of 110 patients developed NIs, with an NIs rate of 9.60%. Among the 110 patients with NIs, lower respiratory tract infections and surgical site infections were predominantly found in 38 (34.55%) and 34 (30.91%) cases, respectively. Other sites of NIs included abdominal infections in 4 cases (3.64%), urinary tract infections in 4 cases (3.64%), ascites infections in 2 cases (1.82%) and bloodstream infection in 1 case (0.91%). Additionally, there were 27 cases (24.55%) of multiple sites of infections. The NIs rates for the training and validation sets were 9.71% and 9.38%, respectively. **Table 1** had displayed the baseline characteristics of study subjects in the training set and validation set.

**Table 1:** Distribution of characteristics of patients in the training set and validation set.

Variable	Training Set			Validation Set		
	Infection group (*n*=74)	Non-Infection group (*n*=688)	*P*	Infection group (*n*=36)	Non-Infection group (*n*=348)	*P*
Gender, n (%)						
Male	23 (31.08)	265 (38.52)	0.210	16 (44.44)	134 (38.51)	0.487
Female	51 (68.92)	423 (61.48)		20 (55.56)	214 (61.49)	
Age (years) ^†^	65.03 ± 12.95	59.58 ± 12.36	<0.001	68.64 ± 11.20	58.18 ± 11.53	<0.001
BMI (kg/m^2^)						
< 25	39 (52.70)	384 (55.81)	0.690	24 (66.66)	212 (60.92)	0.500
≥ 25	35 (47.30)	304 (44.19)		12 (33.33)	136 (39.08)	
Hypertension, n (%)						
Yes	31 (41.89)	207 (30.09)	0.037	16 (44.44)	107 (30.75)	0.094
No	43 (58.11)	481 (69.91)		20 (55.55)	241 (69.25)	
Diabetes, n (%)						
Yes	13 (17.57)	115 (16.72)	0.852	6 (16.67)	53 (15.23)	0.820
No	61 (82.43)	573 (83.28)		30 (83.33)	295 (84.77)	
Preoperative intestinal obstruction, n (%)						
Yes	11 (14.86)	65 (9.45)	0.139	7 (19.44)	29 (8.33)	0.061
No	63 (85.14)	623 (90.55)		29 (80.56)	319 (91.67)	
Smoking, n (%)						
Yes	15 (20.27)	167 (24.27)	0.443	11 (30.56)	262 (75.29)	0.442
No	59 (79.73)	521 (75.73)		25 (69.44)	86 (24.71)	
TNM staging, n (%)						
1-2	32 (43.24)	414 (60.17)	0.005	17 (47.22)	232 (66.67)	0.095
3-4	42 (56.76)	274 (39.83)		19 (52.78)	116 (33.33)	
Preoperative chemotherapy, n (%)						
Yes	8 (10.81)	23 (3.34)	0.005	3 (8.33)	22 (6.32)	0.091
No	66 (89.19)	665 (96.66)		33 (91.66)	326 (93.68)	
Length of preoperative stay, (years) ^†^	5.00 (3.75, 7.00)	5.00 (3.00, 6.00)	0.059	3.00 (2.00, 5.00)	3.00 (2.00, 5.00)	0.878
Peak temperature on the first postoperative day, (°C)	37.68 ± 0.65	37.39 ± 0.50	<0.001	37.71 ± 0.62	37.38 ± 0.52	0.001
Peak temperature on the second postoperative day, (°C)	37.64 ± 0.80	37.17 ± 0.46	<0.001	37.56 ± 0.67	37.17 ± 0.44	0.002
Surgical type, n (%)						
Laparoscopy	86 (56.95)	404 (71.00)	0.001	21 (58.33)	316 (90.80)	<0.001
Open surgery	65 (43.05)	165 (29.00)		15 (41.67)	32 (9.20)	
Combined with other organ removal, n (%)						
Yes	15 (20.27)	43 (6.25)	<0.001	6 (16.67)	13 (3.74)	0.003
No	59 (79.73)	645 (93.75)		30 (83.33)	335 (96.26)	
Enterostomy, n (%)						
Yes	14 (18.92)	20 (2.91)	<0.001	9 (25.00)	9 (2.59)	<0.001
No	60 (81.08)	668 (97.09)		27 (75.00)	339 (97.41)	
Prophylactic application of antimicrobial drugs 30 min before surgery, n (%)						
Yes	69 (93.24)	679 (98.69)	0.004	33 (91.66)	339 (97.41)	0.166
No	5 (6.76)	9 (1.31)		3 (8.33)	9 (2.59)	
Duration of surgery, (min) ^‡^	199.00 (148.75, 250.00)	180.00 (150.00, 214.75)	0.008	177.50 (140.00, 228.75)	175.00 (145.00, 210.00)	0.615
NNIS score, n (%)						
< 2	58 (78.38)	655 (95.20)	<0.001	31 (86.11)	334 (95.98)	0.028
≥ 2	16 (21.62)	33 (4.80)		5 (13.89)	14 (4.02)	
ASA class, n (%)						
<III	43 (58.11)	609 (88.52)	<0.001	24 (66.67)	319 (91.67)	<0.001
≥III	31 (41.89)	79 (11.48)		12 (33.33)	29 (8.33)	
Perioperative blood transfusion, n (%)						
Yes	29 (39.19)	121 (17.59)	<0.001	17 (47.22)	51 (14.66)	<0.001
No	45 (60.81)	567 (82.41)		19 (52.78)	297 (85.34)	
Postoperative complication, n (%)						
Yes	21 (28.38)	14 (2.03)	<0.001	7 (19.44)	3 (0.86)	<0.001
No	53 (71.62)	674 (97.97)		29 (80.56)	345 (99.14)	
Urinary catheter retention time, n (%)						
< 6	26 (35.14)	442 (64.24)	<0.001	13 (36.11)	119 (34.20)	<0.001
≥ 6	48 (64.86)	246 (35.76)		23 (63.89)	229 (65.80)	
Duration of retention of abdominal drains, n (%)						
< 10	42 (56.76)	598 (86.92)	<0.001	24 (66.67)	312 (89.66)	<0.001
≥ 10	32 (43.24)	90 (13.08)		12 (33.33)	36 (10.34)	
Braden score on the operative day, (point) ^‡^	13.53 ± 2.08	15.01 ± 0.90	<0.001	13.50 ± 2.30	15.04 ± 0.79	<0.001
Preoperative WBC (×10^9^/L) ^‡^	5.99 (4.76, 7.83)	5.83 (4.77, 6.95)	0.376	5.73 (5.03, 7.15)	5.57 (4.57, 6.68)	0.162
Postoperative WBC (×10^9^/L) ^‡^	9.14 (6.92, 11.82)	8.97 (7.28, 10.75)	0.379	8.78 (7.21, 10.92)	8.54 (6.63, 11.61)	0.891
Preoperative NEUT% ^‡^	61.65 (51.98, 70.33)	59.45 (53.43, 65.38)	0.249	65.75 (57.98, 73.25)	60.60 (53.33, 66.80)	0.020
Postoperative NEUT%	83.80 (79.25, 88.60)	82.85 (77.50, 86.70)	0.040	86.95 (81.93, 90.13)	83.20 (78.50, 87.20)	0.004
Preoperative Hb (g/L) ^‡^	120.50 (97.25, 138.25)	123.00 (100.00, 140.75)	0.366	101.50 (123.50, 139.00)	126.00 (109.00, 142.00)	0.395
Postoperative Hb (g/L) ^‡^	106.00 (90.00, 122.00)	109.50 (92.00, 125.75)	0.350	109.50 (97.25, 119.25)	114.00 (99.00, 127.00)	0.168
Preoperative PLT (×10^9^/L) ^‡^	245.50 (174.50, 302.25)	257.00 (213.25, 324.75)	0.033	256.00 (199.75, 310.50)	258.00 (204.25, 309.25)	0.971
Postoperative PLT (×10^9^/L) ^‡^	199.00 (140.00, 260.75)	219.00 (178.00, 280.00)	0.011	201.00 (165.00, 268.25)	214.00 (178.00, 264.00)	0.368
Preoperative TP (g/L) ^‡^	67.55 (62.13, 71.45)	67.90 (64.40, 71.38)	0.238	66.75 (59.05, 68.35)	67.45 (64.70, 71.30)	0.186
Postoperative TP (g/L) ^‡^	55.10 (49.55, 58.80)	57.50 (52.90, 61.70)	0.009	52.60 (48.50, 59.53)	56.80 (51.70, 62.60)	0.005
Preoperative Glu (mmol/L) ^‡^	5.35 (4.63, 5.92)	4.99 (4.57, 5.56)	0.015	4.80 (4.35, 6.01)	4.94 (4.47, 5.53)	0.650
Preoperative Cystatin C (mg/L) ^‡^	0.99 (0.85, 1.10)	0.90 (0.80, 1.02)	0.003	0.96 (0.83, 1.20)	0.87 (0.77, 0.98)	0.001
Postoperative Cystatin C (mg/L) ^‡^	0.82 (0.73, 0.95)	0.72 (0.63, 0.83)	<0.001	0.80 (0.69, 0.99)	0.70 (0.62, 0.80)	<0.001
Postoperative PCT (ng/ml) ^‡^	0.40 (0.11, 1.06)	0.14 (0.08, 0.36)	<0.001	0.46 (0.17, 1.63)	0.13 (0.07, 0.32)	<0.001

† mean ± standard deviation (SD).

‡: median (interquartile range).

BMI, body mass index; TNM, Tumor Node Metastasis; ASA, American Society of Anesthesiologists; WBC, White blood cell; NEUT, Neutrophil; Hb, Hemoglobin; PLT, Platelet count; TP, Total protein; Glu, Glucose; PCT, Procalcitonin.

### 3.2 Predictive variables selection

**Figure 2:**
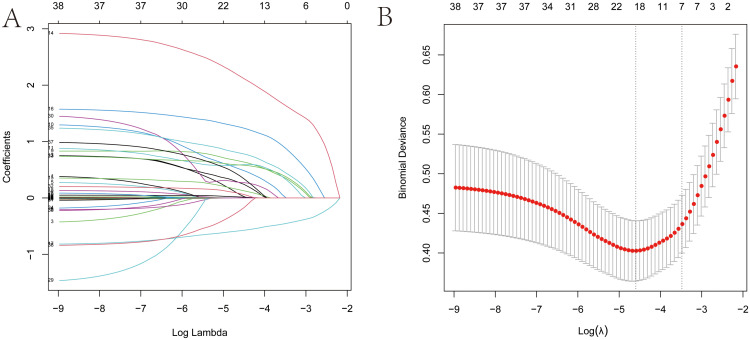
Predictors selection using the least absolute shrinkage and selection operator (LASSO) binary logistic regression model. **(A)** The curve of the coefficient path of variables in the training set. Each curve indicated the trajectory of a variable coefficient change. **(B)** LASSO regression coefficients profiles of variables. The two vertical dashed vertical lines correspond to lambda.min (logarithm of the minimum mean error λ) and lambda.1se (logarithm of the doubled standard error λ). In order to obtain a well-performing and more parsimonious model, seven features with non-zero coefficients were selected as the best variables in this study based on lambda.1se.

We used LASSO regression for the initial screening of predictors of NIs. **Figure 2** present the process of variable screening in LASSO regression. Based on LASSO regression, we ultimately selected seven features as the optimal variables.

### 3.3 Development of the nomogram

**Figure 3:**
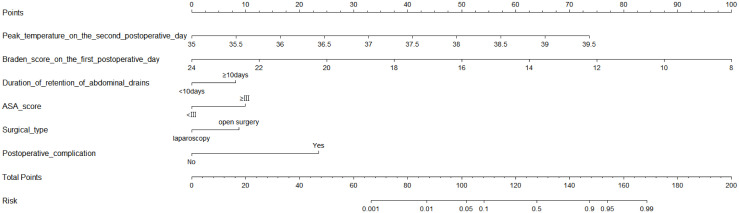
Nomogram for predicting the risk of NIs in patients undergoing colon cancer surgery. ASA, American Society of Anesthesiologists.

**Figure 4:**
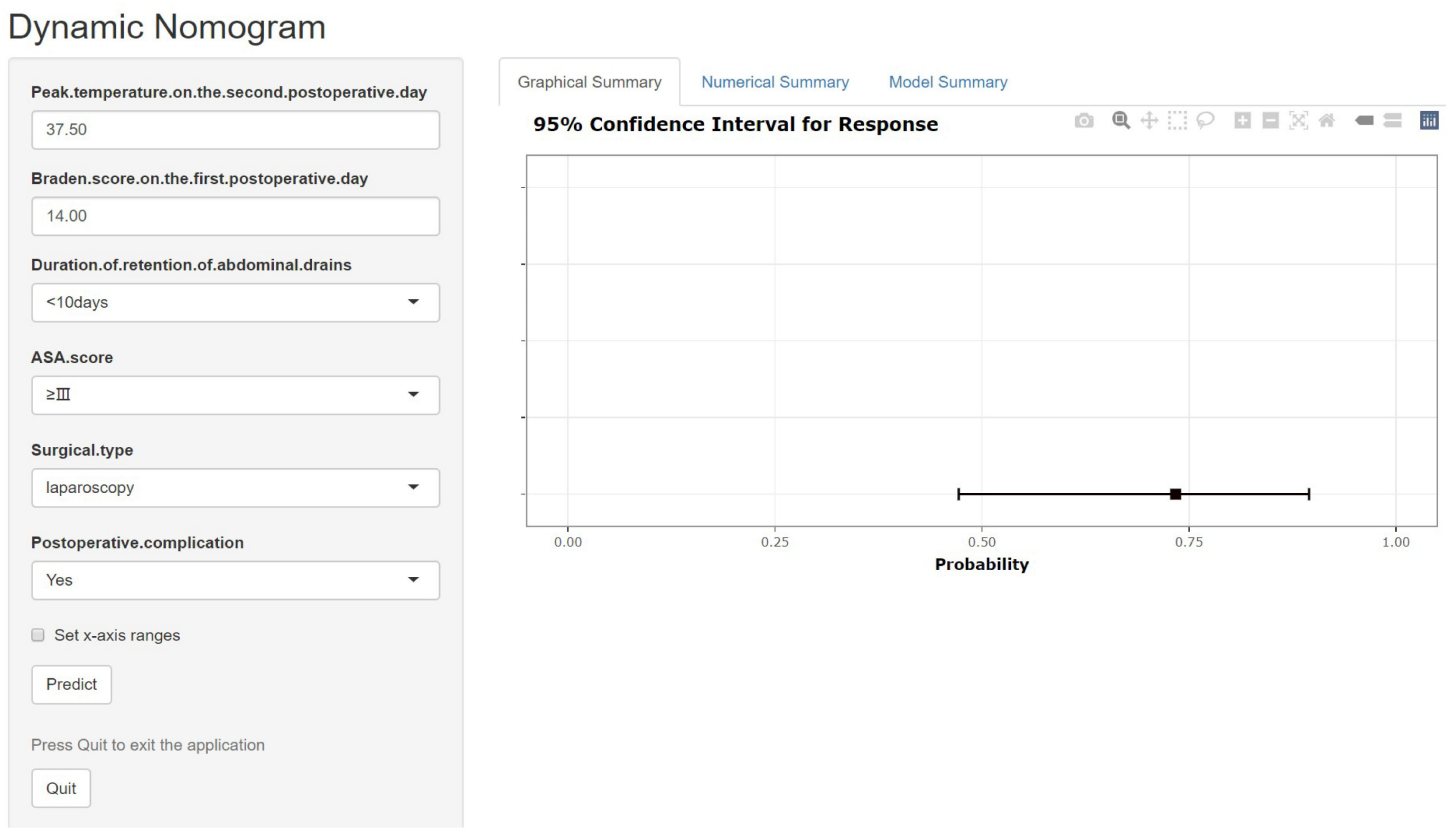
Dynamic nomogram for predicting the risk of NIs in patients undergoing colon cancer surgery. ASA, American Society of Anesthesiologists.

After including the above seven variables in the multivariate logistic regression, a total of six independent predictors were finally obtained (**Table 2**). **Figure 3** showed the nomogram for predicting the risk of NIs in postoperative colon cancer patients constructed on the basis of six independent predictors. In addition, for the convenience of application, we produced a dynamic nomogram(https://jiechangailiexiantu.shinyapps.io/ccDynNomapp/). This web page allows clinicians to automatically calculate the probability of a patient’s risk of postoperative NIs on-line by selecting or entering the values of the predictor variables based on the patient’s actual condition, which is easy to apply and highly efficient. **Figure 4** is a screenshot of an example of the nomogram application. As can be seen from **Figure 4**, the risk of NIs in this colon cancer patient was 73.40%.

**Table 2:** Predictors of nosocomial infection risk in patients.

Variable	*B*	Odds ratio	95% *CI*	*P*
Peak temperature on the second postoperative day	1.842	6.310	3.551-11.211	<0.001
Braden score on the first postoperative day	-0.703	0.495	0.392-0.624	<0.001
Duration of retention of abdominal drains≥10 days	0.914	2.495	1.242-5.015	0.010
ASA class ≥III	1.112	3.041	1.495-6.185	0.002
Surgical type(Open surgery)	0.983	2.672	1.328-5.373	0.006
Postoperative complication	2.649	14.140	5.227-38.252	<0.001
Intercept	-62.960	<0.001	–	<0.001

CI, confidence interval; ASA, American Society of Anesthesiologists.

### 3.4 Verification of the nomogram

**Figure 5:**
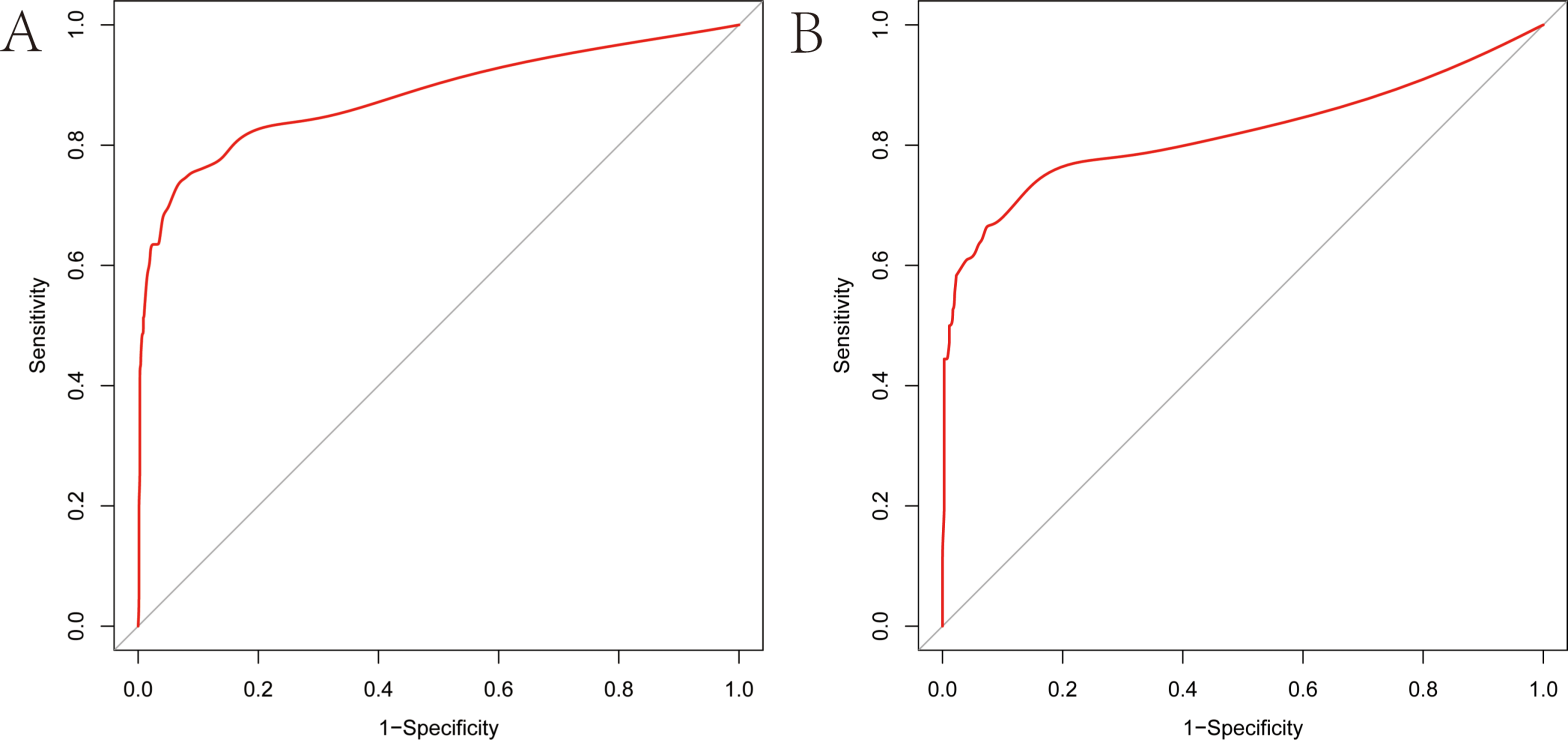
Receiver operating characteristic (ROC) curves of the nomogram **(A)** ROC curve of the training set. **(B)** ROC curve of the validation set. The vertical axis represents the true positive rate, and the horizontal axis represents the false positive rate. The higher the convexity of the red curve, the higher the AUC is demonstrated.

**Figure 6:**
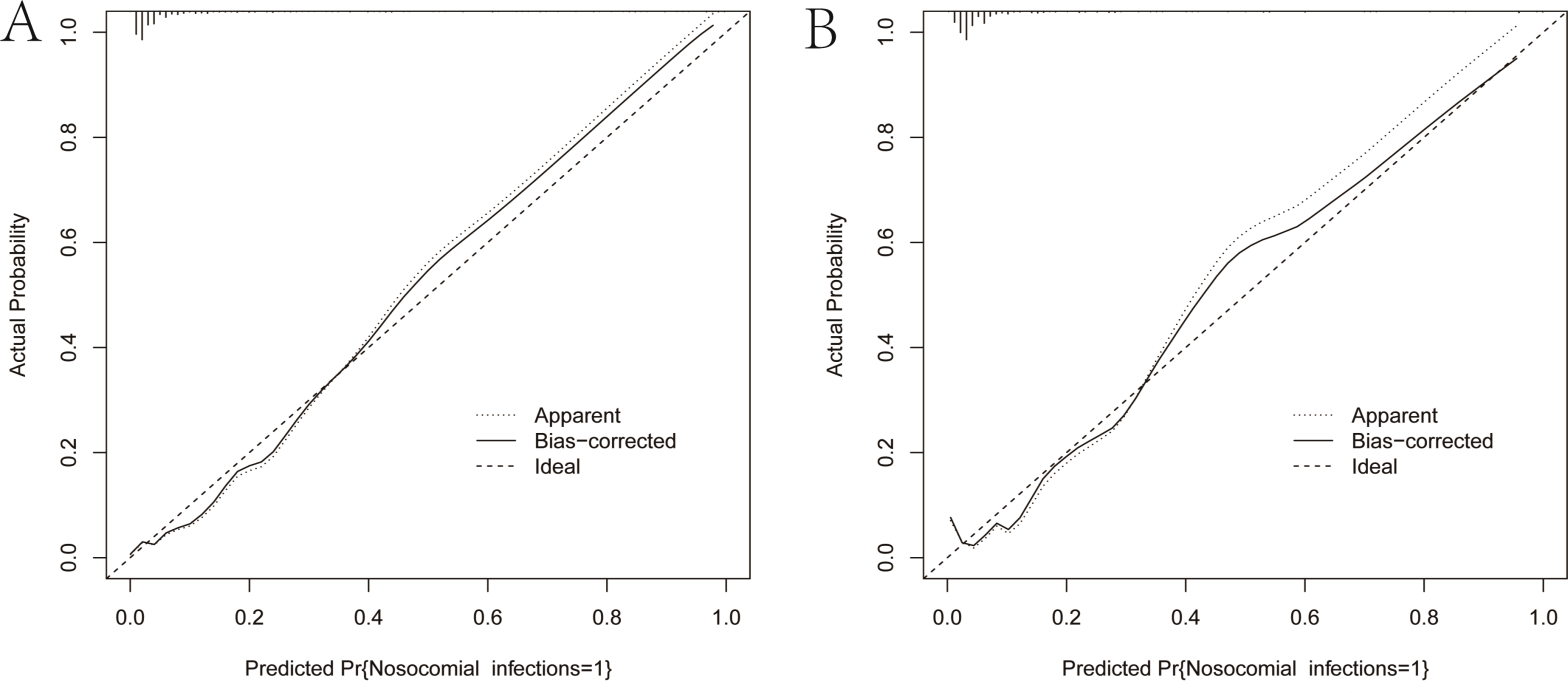
Calibration curves of the nomogram. **(A)** Calibration curve of the training set. **(B)** Calibration curve of the validation set. The solid black line is the result of bias correction by bootstrap resamples (1000 repetitions). The closer the solid black line is to the diagonal dashed line, the better the calibration of the nomogram.

**Figure 7:**
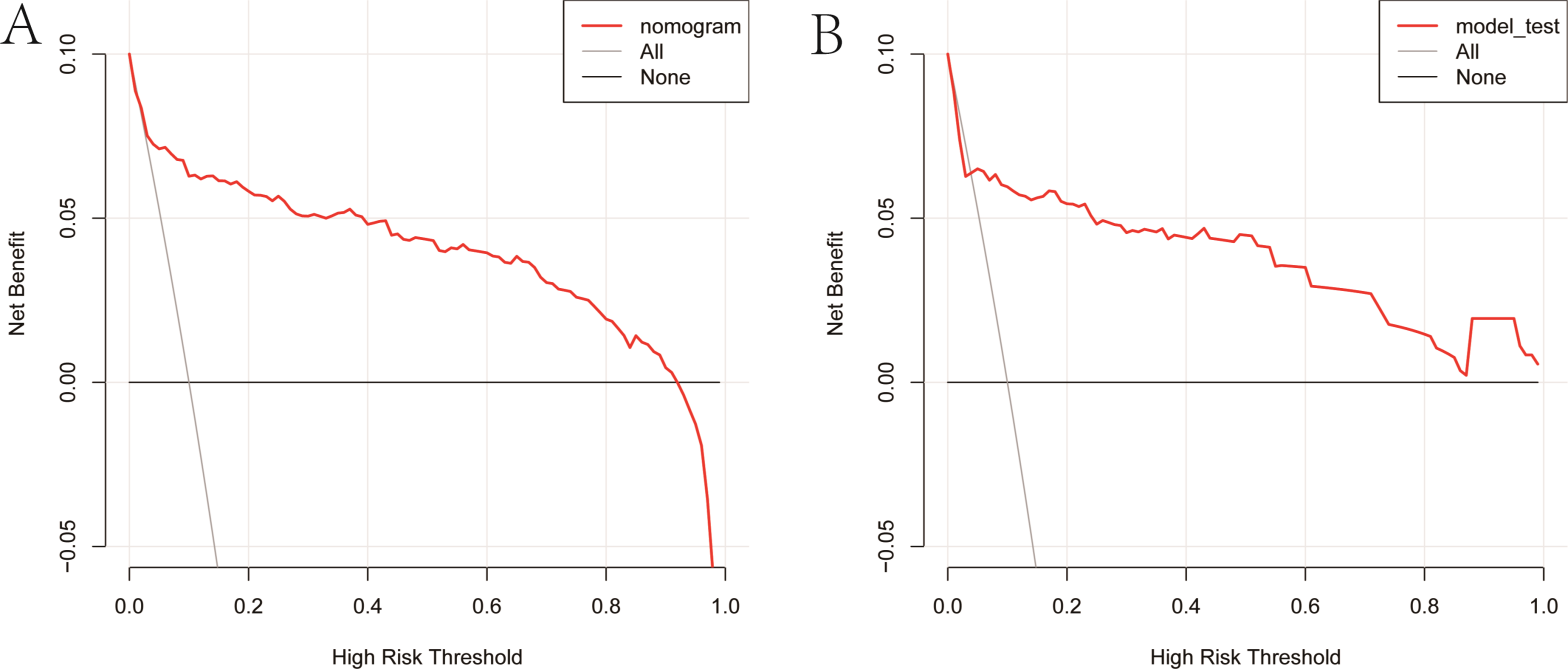
Clinical decision curves of the nomogram. **(A)** Clinical decision curve of the training set. **(B)** Clinical decision curve of the validation set. The gray diagonal line in the Figure represented the intervention performed on all patients. When the red curve did not coincide with the gray diagonal line and lies above the black horizontal line, it indicated that the nomogram has a net benefit.

**Figure 8:**
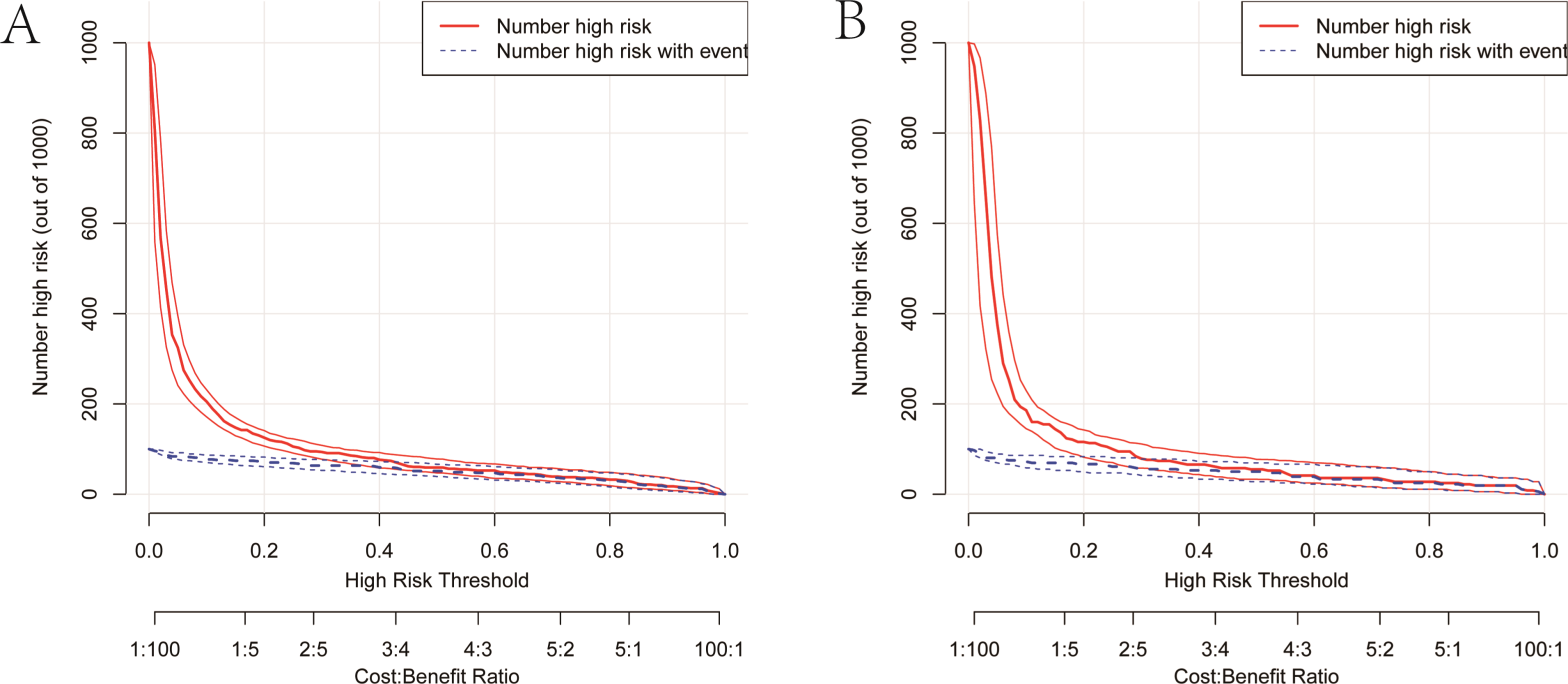
Clinical impact curves of the nomogram. **(A)** Clinical impact curve of the training set. **(B)** Clinical impact curve of the validation set. Among 1,000 patients, the red solid line displays the total number of people considered high-risk at each risk threshold. The blue dashed line represents the true NIs patients among them.

As illustrated in **Figure 5**, The AUC of the training set and validation set were 0.881 (95% *CI*: 0.856~0.903) and 0.813 (95% *CI*: 0.770~0.851), respectively, indicating that the model has good discriminative ability. **Figure 6** showed the calibration curves of the training and validation sets. The bias correction curves in both sets were close to the ideal curves. In addition, the *P* of the Homer Lemeshow test results for the training and validation sets were 0.490 and 0.179, respectively. All the above results showed that the model is well calibrated. The DCA for the training and validation sets were represented in **Figure 7**. The DCA in our study revealed good net benefits of the nomogram in both the training and validation sets. **Figure 8** demonstrated the CIC of the training and validation sets. As shown by the curves in the Figure, the number of patients with NIs predicted by the nomogram was close to the number of patients with NIs that actually occurred. The CIC showed that the nomogram model has good clinical utility.

## 4 Discussion

Delayed diagnosis of NIs leads to prolonged hospitalization and recovery time for patients, and even re-operation. NIs also bring unnecessary medical expenses to patient families, and waste medical resources. Therefore, to achieve better clinical outcomes for colon cancer patients, it is necessary to screen out high-risk patients for NIs in advance and take preventive measures as early as possible to reduce the incidence of NIs. It is worth noting that the dynamic nomogram developed in this study resembles a network calculator. In this dynamic nomogram, we can enter the values of the predictors on the page to automatically calculate the predicted values of the ending events (21).

After analysis, this study concluded that peak temperature on the second postoperative day, Braden score on the first postoperative day, duration of retention of abdominal drains≥10days, ASA class ≥III, surgical type and postoperative complication were significant predictors of NIs. Nomograms generated using the above predictors have good discriminatory power, calibration and clinical validity, and to some extent it can predict the risk of NIs after resection of colon cancer.

Regular postoperative monitoring of patients’ vital signs can help healthcare professionals detect some postoperative infectious complications in a timely manner. Among them, temperature change is the first signal of infection perceived by the patient, and an elevated trend suggests that the patient may have potential inflammation in the organism (22). The postoperative body temperature data of colon cancer patients is easily obtainable in clinical setting. The nomogram of this study shows that the peak temperature on the second postoperative day is an essential factor affecting the occurrence of NIs in colon cancer patients, which further emphasizes the importance of monitoring patients’ postoperative temperatures on schedule. Zheng S et al. (22) reported that the average body temperature on the three postoperative day of patients exceeded 37°C may be a critical sign of surgical site infection.

Postoperative complications were shown to be one of the risk factors for NIs in patients in this study. Colon cancer surgery involves anastomosis between intestinal incisions. Anastomotic leakage can likely occur postoperatively if the patient has problems such as poor overall preoperative nutritional status, insufficient intestinal cleanliness, and poor blood flow to the postoperative intestinal anastomosis. Anastomotic leakage is a potentially serious complication that occurs after colon cancer surgery, which will increase the tumor recurrence rate and mortality rate of patients (23, 24). A meta-analysis by Lawler J. et al. showed that anastomotic leakage had a negative impact on the overall survival of colorectal cancer patients (25).

The surgical procedures performed on colon cancer patients in this study included open and laparoscopic. A meta-analysis synthesizing the results of several randomized controlled studies showed that laparoscopic colectomy has similar disease-free and overall survival rates to open colectomy and that the procedure is safe (25–27). Four randomized controlled trials on colorectal cancer surgery have confirmed that laparoscopic surgery has significant advantages over open surgery in terms of less intraoperative blood loss, less postoperative pain, faster recovery of intestinal function, and shorter length of hospital stay (28). The results of our study showed that the rate of NIs was significantly lower in laparoscopic surgery than in the group of patients who underwent open surgery, which is in line with the results of previous studies (29–33). This is further evidence that expanding the adoption of laparoscopic surgery may reduce the rate of NIs in colon cancer surgery.

In this study, the level of postoperative Braden score was inversely associated with the risk of NIs in colon cancer patients. The Braden Scale is a tool recommended by the U.S. Agency for Healthcare Policy Research in 1987 to predict the risk of pressure ulcers. Many studies in recent years have confirmed that the Braden Scale has other uses. Lovicu E et al. (34) found that admission Braden score was inversely associated with the risk of in-hospital mortality in COVID-19 patients (OR=0.76). Cohen RR et al. found that lower Braden scores predicted postoperative complications (OR=1.30) in elderly surgical patients (34). Although the Braden Scale is a widely used tool for routine nursing assessment of pressure ulcer events, it has been proposed by a previous researcher to be used as a frailty assessment tool because it simultaneously assesses several frailty-related indicators, such as nutrition, cognition, and function (35). The lower the Braden score of a colon cancer patient means that the patient’s physical mobility and nutritional intake, among other things, are likely to be poorer, which can lead to a low level of physical resistance and an increased chance of infection.

In this study, ASA class ≥ III (OR=3.041) was a predictor of NIs after colon cancer surgery, which was similar to the findings of Yang J et al. (36). The ASA class is a method of representing patient operative risk on a scale of I-VI (37). The ASA class ≥ III often implies that the patient is in poorer health and at higher risk of NIs. Therefore, clinical healthcare professionals should actively adjust the physical status of colon cancer patients, and adopt multidisciplinary consultation when necessary in order to minimize the risk of NIs in patients (22).

Similar to the results of this study, several previous studies have reported a correlation between indwelling prophylactic abdominal drains and the development of retrograde infectious complications, which may be related to the retrograde entry of pathogenic bacteria into the abdominal cavity through the line (38, 39). Clinical practice guidelines in the United States clearly state that it is recommended that routine use of abdominal drains should be avoided after colorectal surgery, given that there are no data to support the benefit of routine use of abdominal drains in the identification and prophylactic treatment of anastomotic fistulas, but rather the potential to lead to the development of drainage-associated complications such as extra-intestinal fistulas (40).

The strength of this study is that the constructed dynamic nomogram is based on clinically available predictors that can be used to guide healthcare professionals in developing strategies for NIs prevention and control. Compared with static nomograms, dynamic nomograms interactive interface is more convenient for clinicians to make personalized diagnosis and treatment decisions, which can simplify the complexity of the clinical practice of nomograms, and improve the efficiency of the use of nomograms.

There are some limitations to our study. First, the nomogram was not validated using data from other hospitals, which may limit the generalizability of the nomogram. Second, we only collected and compared patients’ NIs during their stay in the hospital, but failed to follow them long-term after discharge. Therefore, multicenter, large sample as well as prospective analyses should be conducted in the future.

## 5 Conclusion

We developed a dynamic nomogram of NIs risk with good discrimination, calibration, and clinical validity. This web-based online risk calculator may help healthcare professionals to identify patients at high risk of NIs early.

## Data Availability

The raw data supporting the conclusions of this article will be made available by the authors, without undue reservation.
